# Downregulation of Lipid Phosphate Phosphatase 3 Correlates With Tumor-Infiltrating Immune Cells in Oral Cancer

**DOI:** 10.7759/cureus.23553

**Published:** 2022-03-27

**Authors:** Supriya Vishwakarma, Deepti Joshi, Ritu Pandey, Saikat Das, Sramana Mukhopadhyay, Renu Rai, Ritu Singhal, Neelkamal Kapoor, Ashok Kumar

**Affiliations:** 1 Department of Biochemistry, All India Institute of Medical Sciences, Bhopal, IND; 2 Department of Pathology and Laboratory Medicine, All India Institute of Medical Sciences, Bhopal, IND; 3 Department of Radiotherapy, All India Institute of Medical Sciences, Bhopal, IND; 4 Department of Oncology, Jawaharlal Nehru Cancer Hospital and Research Centre, Bhopal, IND; 5 Department of Pathology, Jawaharlal Nehru Cancer Hospital and Research Centre, Bhopal, IND

**Keywords:** sphingosine kinase 2, lipid phosphate phosphatase 3, sphingosine kinase 1, sphingosine-1-phosphate, oral squamous cell carcinoma

## Abstract

Background

Sphingosine-1-phosphate (S1P) is a potent oncogenic lipid. Intracellular levels of S1P are tightly regulated by eight S1P-metabolizing enzymes. S1P synthesis is catalyzed by two sphingosine kinases, i.e., sphingosine kinase 1 (SphK1) and sphingosine kinase 2 (SphK2). Five lipid phosphatases (two S1P phosphatases and lipid phosphate phosphatases (LPPs) 1, 2, and 3) reversibly convert S1P back to sphingosine. Previously, we have determined the mRNA expression profile of eight S1P-metabolizing enzymes in tumor tissues and adjacent normal tissues from oral squamous cell carcinoma (OSCC) patients. Except for SphK1, the role of S1P-metabolizing enzymes in OSCC has been poorly studied.

Methods

We have determined the protein expression of four S1P-metabolizing enzymes (SphK1, SphK2, sphingosine-1-phosphate phosphatase 1 (SGPP1), and lipid phosphate phosphatase 3 (LPP3)) by immunohistochemistry (IHC) in tumor tissues of 46 OSCC patients. Six subjects with non-dysplastic oral mucosa were also included in the study. The immunoreactivity score (IRS) was calculated for each protein in every subject. Further, we determined the associations of expression of S1P-metabolizing enzymes with clinicopathological features of OSCC patients.

Results

We demonstrate the low IRS for SphK2 and LPP3 in OSCC tumors. Importantly, expression of SphK2 and LPP3 was downregulated in malignant epithelial cells compared to non-malignant mucosa. Further, LPP3 expression negatively correlated with tumor‑node‑metastasis (TNM) staging of patients (r = −0.307, p = 0.043). Importantly, expression of LPP3 in tumors was found to be an independent predictor of perinodal extension (b = −0.440, p = 0.009), lymphovascular invasion (b = −0.614, p < 0.001), lymph node ratio (b = 0.336, p = 0.039), and TNM staging (b = −0.364, p = 0.030).

Conclusion

Taken together, our data show that expression of SphK2 and LPP3 is decreased compared to normal mucosa. Thus, the S1P signaling pathway could represent a potential therapeutic target.

## Introduction

Head and neck cancer (HNC) is the sixth most common cancer globally [1]. As per the GLOBOCAN 2020 data, the age-standardized rate per 100,000 for cancer of the lip and oral cavity in males is twice in developing countries than in developed countries [1]. The majority of the oral cavity, oropharyngeal, hypopharyngeal, and laryngeal cancers are squamous cell carcinoma (SCC) in histology. Squamous cell cancer of the lip and oral cavity are referred to as oral squamous cell carcinoma (OSCC), which is the most common cancer in Indian men and accounts for one-sixth of all cancers [2]. The global incidence of OSCC is more than 350,000 [1], out of which one-fourth of the cases (75,000) is contributed by the Indian sub-continent [2]. Rapid advances in diagnosis, availability of prognostic markers, and early management protocols have not increased the five-year survival rate.

Management of OSCC includes surgery, chemotherapy, radiotherapy, and combination therapy depending on site, age, and tumor‑node‑metastasis (TNM) staging. Conventional therapies are non-selective and often remove normal tissues in the complex orofacial region [3]. Molecularly targeted therapies offer new treatment options even for patients who are unable to tolerate chemotherapy or radiation therapy. Though epidermal growth factor receptor antibody (cetuximab) offers clinical gain; however, intrinsic resistance and the development of acquired resistance to cetuximab are well-recognized phenomena in OSCC [4]. Therefore, identifying novel oncogenic pathways in OSCC could lead to the development of targeted therapy combinations and overcome drug resistance.

Sphingosine-1-phosphate (S1P), a potent sphingolipid metabolite, mediates its actions by binding to its cognate G-protein coupled receptors, termed S1P receptors [5]. S1P has diverse pleiotropic biological functions, including lymphocyte trafficking, vascular angiogenesis, and muscle regeneration [5]. S1P signaling regulates several processes integral to carcinogenesis, including inflammation, cancer initiation, progression, invasion, metastasis, and drug and radiation resistance [5-7]. However, the role of S1P signaling in oral carcinogenesis is not fully understood.

Eight metabolic enzymes tightly regulate intracellular S1P levels. S1P is synthesized intracellularly by two sphingosine kinases (SphKs), namely, sphingosine kinase 1 (SphK1) and sphingosine kinase 2 (SphK2). Both the isoforms catalyze the phosphorylation of sphingosine to form S1P [8]. Further, the catabolism of S1P is regulated by six enzymes. Out of six S1P-catabolizing enzymes, two are S1P phosphatases, namely, sphingosine-1-phosphate phosphatase 1 (SGPP1) and sphingosine-1-phosphate phosphatase 2 (SGPP2), that dephosphorylate S1P back to sphingosine [7-10]. In addition, broad-specificity lipid phosphate phosphatases (LPPs) 1, 2, and 3 can also dephosphorylate S1P [11,12]. Importantly, S1P lyase irreversibly degrades S1P into a hexadecenal and ethanolamine phosphate [5].

SphK1 is linked with pro-survival and cell maintenance functions [8]; however, its second isoform, SphK2, has been shown to perform dual tasks, i.e., a protective role in cell maintenance as well as its pro-apoptotic functions [8]. SphK1 promotes tumor progression, invasion, metastasis, and chemoresistance and is a well-established oncogene [8]. SphK1 is abundantly expressed in various types of cancers, including OSCC, and is a marker for poor prognosis [8]. A low increase in SphK2 levels (2.5 fold), compared to the levels in corresponding normal tissue, could potentially promote cell proliferation and neoplastic transformation. In contrast, high levels of SphK2 are associated with pro-apoptotic signaling, predicted to be through the unique BH3 pro-apoptotic domain that dominates over the proliferative SphK2/S1P response [13].

Except for SphK1, the role of S1P-metabolizing enzymes in OSCC has not been fully understood. Earlier, we reported that in approximately two-thirds of OSCC patients studied, mRNA expression of SphK2 and lipid phosphate phosphatase 3 (LPP3) was significantly downregulated in tumor tissues, compared with the same patient’s adjacent normal tissue [14]. Since no information is available regarding immunohistochemical localization of SphK2, SGPP1, and LPP3 in OSCC, thus, in the present study, we have analyzed the expression of SphK1, SphK2, SGPP1, and LPP3 in tumor tissues from OSCC patients and benign oral mucosa by immunohistochemistry (IHC).

The tumor microenvironment (TME) of head and neck squamous cell carcinomas (HNSCCs) consists of many different subsets of cells, including cells of the immune system that infiltrate the tumors [15]. Emerging evidence from recent studies suggests that tumor-infiltrating immune cells (TIICs) play an essential role in immunogenic cytotoxicity and tumor response to establish immune tolerance [15,16]. TIICs, especially tumor-infiltrating lymphocytes (TILs), are critical prognostic features of HNSCC tumors. The presence of effector immune cells in the TME is associated with a better clinical response to chemo-radiation [15,17]. S1P signaling has emerged as a central regulator of the trafficking of immune cells, including lymphocytes, natural killer (NK) cells, neutrophils, dendritic cells (DCs), and macrophages [7]. However, the role of S1P-metabolizing enzymes in the regulation of TIICs in HNSCC has not been elucidated.

Here, we have determined the association of expression of S1P-metabolizing enzymes with clinicopathological attributes of OSCC patients. In this study, we demonstrated that the expression of SphK2 and LPP3 was downregulated in OSCC tumors compared to non-malignant mucosa. Further, LPP3 expression negatively correlated with TNM staging of patients. In The Cancer Genome Atlas (TCGA) analysis, LPP3 expression correlated with the infiltration levels of B cells, regulatory T cells (Tregs), neutrophils, macrophages, and DCs in HNSCC tumors.

The pre-print of this paper is available on Research Square (DOI: 10.21203/rs.3.rs-1043910/v1).

## Materials and methods

Subjects and tissue collection

A total of 46 patients with OSCC (International Classification of Diseases 10: C00-08) over the age of 18 years, who had not undergone radiotherapy and chemotherapy, were enrolled in the study at Jawaharlal Nehru Cancer Hospital and Research Centre, Bhopal. In addition, six subjects with non-dysplastic oral mucosa were also included in the study. Informed consent was obtained from all the participants. Data on the initial diagnosis and clinical history of patients were recorded. The above project was approved by the Institutional Human Ethics Committee, All India Institute of Medical Sciences, Bhopal (IHEC-LOP/2016/EF0001).

Western blotting

Tumor and adjacent normal tissues (at least 1 cm away from tumor margin) were collected in microcentrifuge tubes and flash-frozen in liquid nitrogen immediately after surgical resection. Approximately 50 mg of tissue was homogenized in a lysis buffer containing 150 mM NaCl, 1 mM MgCl2, 1 mM NaF, protease inhibitor cocktail, and 1% triton-X 100. Total protein was quantitated using Bradford’s reagent (Bio-Rad Laboratories, Hercules, CA). Forty micrograms of protein was separated on 10% sodium dodecyl sulfate-polyacrylamide gel electrophoresis (SDS-PAGE). Proteins were electrotransferred to the nitrocellulose membrane. Non-specific sites were blocked by 10% non-fat milk in Tris-buffered saline containing 0.05% Tween 20. Membranes were incubated with primary antibodies, human SphK2 (Cat# SAB1300017) (Sigma-Aldrich, St. Louis, MO), human SGPP1 (Biorbyt, Cambridge, UK), human LPP3 (Cat# HPA028892, Sigma-Aldrich), and beta-actin (Sigma-Aldrich). After washing, membranes were incubated with horseradish peroxidase-conjugated secondary antibodies. Immune complexes were recognized with enhanced chemiluminescence substrate (Thermo Fisher Scientific, Waltham, MA). Signals were captured with Chemidoc (Syngene, Cambridge, UK). The intensity of bands was quantitated with ImageJ software (National Institutes of Health, Bethesda, MD).

Immunohistochemistry

All immunohistochemical analyses were carried out using the Vectastain Universal Elite ABC Kit (Vector Laboratories, Burlingame, CA). Tissue sections (4-5 microns) were mounted on glass slides using 3-aminopropyltriethoxysilane (APES) solution (1:20 in methanol) and deparaffinized in a hot-air oven for 10 minutes. Further deparaffinization was done with xylene and rehydrated with graded alcohol. Endogenous peroxidase activity was blocked with 3% hydrogen peroxide in methanol for 20 minutes, followed by phosphate-buffered saline (PBS) wash thrice. Antigen retrieval was done by immersing slides in 10 mM sodium citrate buffer (pH 6.0) and placed in a microwave for 20 minutes to enhance antigen exposure. Then, blocking was done with normal goat serum for one hour, followed by incubation with the primary antibody at 1:100 dilution in blocking buffer (normal goat serum) overnight at 4°C. Rabbit polyclonal antibodies against human SphK1 (Cell Signaling Technology, Danvers, MA), human SphK2 (Cat# SAB1300017) (Sigma-Aldrich), human SGPP1 (Biorbyt, Cambridge, UK), and human LPP3 (Cat# HPA028892, Sigma-Aldrich) were used as the primary antibodies. Immunostaining was developed using the Vectastain ABC reagents (Vector Laboratories), according to the biotinylated immune-peroxidase technique followed by 3,3′-diaminobenzidine (DAB substrate) staining. Tissue sections were counterstained with hematoxylin.

Assessment of immunohistochemical staining

Immunoreactivity for SphK1, SphK2, SGPP1, and LPP3 was semi-quantitatively esti­mated by combining intensity and percentage of positive tumor cells under the microscope. The immunostaining results were optimized and judged by two independent pathologists according to the following standards. At least five fields were examined in each section. Based on all the field views, the immunoreactivity score (IRS) was calculated for each slide. The staining intensity was scored as follows: 0 (negative staining), 1 (mild staining), 2 (moderate staining), and 3 (strong staining). The percentage of stained cells was classified as follows: 0 (<10%), 1 (10-50%), 2 (51%-80%), and 3 (>81%). The final IRS for each protein, ranging from 0 to 9, was obtained by multiplying the percentage of positive cells and the intensity score. Patients with a final IRS score were classified as low (1-4) and high (≥5). TIICs were counted in the high-power field of hematoxylin and eosin-stained sections of OSCC tumors. Immunostaining was performed with consecutive sections with all antibodies. Results were interpreted by at least two pathologists.

Association of mRNA expression of S1P-metabolizing genes and immune cells

Tumor Immune Estimation Resource (TIMER) is a web server for the comprehensive analysis of TIICs [18]. The group has launched the updated and enhanced version of the web server, TIMER 2.0, which can be used to systematically analyze immune infiltration across diverse cancer types [19]. TIMER 2.0 (http://timer.cistrome.org/) provides a more robust estimation of immune infiltration levels for TCGA or user-provided tumor profiles using six algorithms [19]. Association between mRNA expression of S1P-metabolizing enzymes and TIICs (B cells, CD4+ T cells, CD8+ T cells, regulatory T cells (Tregs), neutrophils, macrophages, DC, and NK cells) was analyzed by the "Immune" module of TIMER 2.0, where gene expression profile from human papillomavirus (HPV)-negative (n = 422) and HPV-positive patients (N = 98) and HNSCC patients (N = 522) was used. Gene_DE module of TIMER 2.0 was used to study the differential expression between tumor and adjacent normal tissues for phosphatidic acid phosphatase type 2B (PPAP2B) across all TCGA tumors.

Knockdown of LPP3 using lentiviral short hairpin RNA (shRNA)

The human cervical cancer cell line, Ca Ski (ATCC-CRL-1550™), was obtained from the American Type Culture Collection (Manassas, VA). The cell line was maintained in DMEM-F12 (Gibco, Grand Island, NY), supplemented with 10% fetal bovine serum (Gibco), 100 units/ml of penicillin and streptomycin (Gibco), and 0.4 µg/ml hydrocortisone (MP Biomedicals India Pvt Ltd, Mumbai, India). The cell line was cultured in a humidified atmosphere at 37°C and 5% CO2. To knock down the expression of PPAP2B gene coding for LPP3, 0.3 million Ca Ski cells were seeded in six well plates; upon reaching 60-70% confluency, cells were transfected with 4 µg of shControl and pLKO.1-shPPAP2B (TRC No. TRCN0000050457, Sigma-Aldrich) using Fugene (Promega, Fitchburg, WI) at 1:2 ratio (DNA Plasmid, Fugene). After 60 hours of incubation at 37°C and 5% CO2, cells were retrieved for western blot analysis.

Statistical analysis

Data were entered in SPSS software, version 21.0 (IBM Corp., Armonk, NY). Descriptive analysis was done by SPSS software. Patient data, including age, gender, tobacco chewing, type of tobacco/betel nut consumed, alcohol consumption, site of the tumor, history of smoking, tumor volume, TNM staging, lymph node ratio (LNR), lymphovascular invasion (LVI), and perinodal extension (PNE) were entered into the database. The difference in the mean was determined by Student’s t-test. To determine the association of IHC expression in OSCC of various S1P-metabolizing enzymes, data from patients were categorized into two groups: low expression and high expression. The association of IRS with clinicopathological features was examined by Spearman’s rank test. To determine clinicopathological attributes as an independent predictor of expression of S1P-metabolizing enzymes or vice versa, multivariate linear regression analysis was performed.

## Results

Patients characteristics

The median age of the OSCC patients enrolled in the study was 46 years (25-75 years), and approximately two-thirds (67.40%) of subjects were males. More than half (58.7%) patients were younger than 50 years of age. Three-fourths of the subjects had a history of tobacco/betel nut chewing (78.30%). Approximately more than one-fourth of the subjects had a history of alcohol consumption or smoking (Table 1). Buccal mucosa was the most common (50%) site for oral cancer in the subjects, followed by the tongue (26%) (Table 1). More than three-fourths (78.2%) were presented in the advanced stages (stage III + IV) of OSCC (Table 1); however, no distant metastasis was observed in our study. Histologically, most of the OSCC tumors (82.6%) were well-differentiated squamous cell carcinoma (WDSCC) (Table 1).

**Table 1:** Frequency of SphK1 and SphK2 IRS score. The frequency of immunoreactivity score (IRS) of sphingosine kinase 1 (SphK1) and sphingosine kinase 2 (SphK2) has been calculated with different clinicopathological attributes of oral squamous cell carcinoma patients. Tumor-node-metastasis (TNM) is grouped into two (Group I and Group II), whereas histology has been divided as moderately differentiated squamous cell carcinoma (MDSCC), well-differentiated squamous cell carcinoma (WDSCC), and poorly differentiated squamous cell carcinoma (PDSCC).

S. No.	Groups	Total (n = 46)	SphK1 expression		SphK2 expression	
			Low	High	Low	High
1	Age					
	<50 years	27	14 (51.9%)	13 (48.2%)	23 (85.2%)	04 (14.8%)
	>50 years	19	08 (42.1%)	11 (57.9%)	15 (78.9%)	04 (21.1%)
	P-value	NS				
2	Sex					
	Male	31	15 (48.4%)	16 (51.6%)	23 (74.2%)	08 (25.8%)
	Female	15	07 (46.7%)	08 (53.3%)	15 (100.0%)	00 (00.00%)
	P-value		NS		0.030	
3	Tumor size					
	T1-T2	13	07 (53.8%)	06 (46.2%)	11 (84.6%)	02 (15.4%)
	T3-T4	31	14 (45.2%)	17 (54.8%)	26 (83.9%)	05 (16.1%)
	P-value		NS			
4	Lymph nodes					
	N0	19	10 (52.6%)	09 (47.4%)	14 (73.7%)	05 (26.3%)
	N1-N3	25	11 (44.0%)	14 (56.0%)	23 (92.0%)	02 (08.0%)
	P-value		NS			
5	Distant metastasis					
	M0	44	21 (47.2%)	23 (52.8%)	37 (84.1%)	07 (15.9%)
	M1	00	0	0	0	0
	P-value		NS			
6	Smoking status					
	Yes	10	05 (50.0%)	05 (50.0%)	06 (60.0%)	04 (40.0%)
	No	36	17 (47.2%)	19 (52.8%)	32 (88.9%)	04 (11.1%)
	P-value		NS		0.033	
7	Alcohol status					
	Yes	10	05 (50.0%)	05 (50.0%)	08 (80.0%)	02 (20.0%)
	No	32	13 (40.6%)	19 (59.4%)	27 (84.4%)	05 (15.6%)
	P-value		NS			
8	Tumor volume					
	Less than 4 cm3	20	08 (40.0%)	12 (60.0%)	15 (75.0%)	05 (25.0%)
	More than 4 cm3	24	13 (54.2%)	11 (45.8%)	22 (91.7%)	02 (08.3%)
	P-value		NS			
9	Peri-nodal extension (PNE) involvement					
	Yes	11	06 (54.5%)	05 (45.5%)	10 (90.9%)	01 (09.1%)
	No	33	15 (45.5%)	18 (54.5%)	27 (81.8%)	06 (18.2%)
	P-value		NS			
10	Lymphovascular invasion (LVI)					
	Yes	03	01 (33.3%)	02 (66.7%)	03 (100.0%)	00
	No	38	19 (50.0%)	19 (50.0%)	32 (84.2%)	06 (15.8%)
	P-value		NS			
11	Lymph-node ratio (LNR)					
	Less than < 0.1	29	15 (51.7%)	14 (48.3%)	24 (82.8%)	05 (17.2%)
	More than > 0.1	15	06 (40.0%)	09 (60.0%)	13 (86.7%)	02 (13.3%)
	P-value		NS			
12	Tumor-node-metastasis (TNM) staging					
	Group 1 (Stage I + II)	08	05 (62.5%)	03 (37.5%)	06 (75.0%)	02 (25.0%)
	Group 2 (Stage III + IV)	36	16 (44.4%)	20 (55.6%)	31 (86.1%)	05 (13.9%)
	P-value		NS			
13	Tumor site					
	Buccal mucosa	23	12 (52.2%)	11 (47.8%)	20 (87.0%)	03 (13.0%)
	Tongue	12	05 (41.7%)	07 (58.3%)	09 (75.0%)	03 (25.0%)
	Alveolus	7	03 (42.9%)	04 (57.1%)	06 (85.7%)	01 (14.3%)
	Lip	4	02 (50.0%)	02 (50.0%)	03 (75.0%)	01 (25.0%)
	P-value		NS			
14	Histology					
	WDSCC	38	18 (47.4%)	20 (52.6%)	31 (81.6%)	07 (18.4%)
	MDSCC	08	04 (50.0%)	04 (50.0%)	07 (87.5%)	01 (12.5%)
	PDSCC	00	00	00	00	00
	P-value		NS			

Validation of antibodies

**Figure 1:**
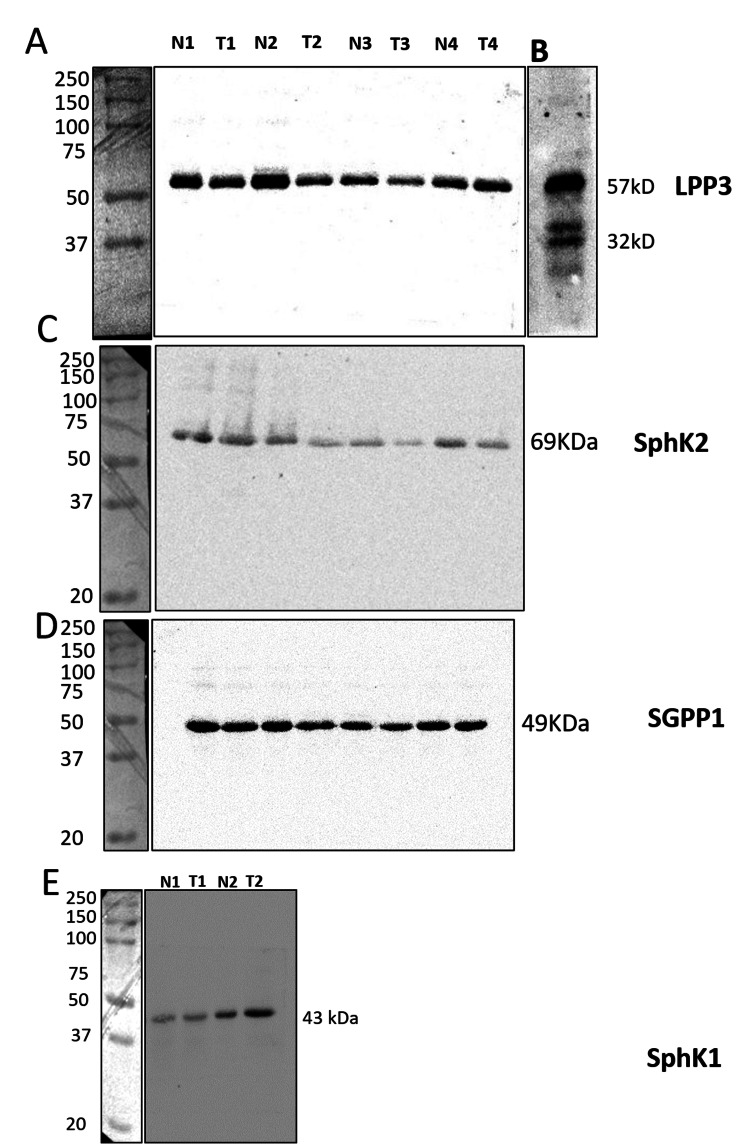
Western blotting for LPP3, SphK2, SGPP1, and SphK1 in the OSCC tumors and normal tissues. Western blotting for LPP3 (A-B), SphK2 (C), and SGPP1 (D) was performed in tumor tissues (T1, T2, T3, and T4) and adjacent normal tissues (N1, N2, N3, and N4) from the same patients. (E) Immunoblotting was performed in two sets of samples (N1, N2, T1, and T2). Photographs of the protein ladder were taken using Chemidoc under white light and were aligned with the immunoblots. Antibodies detected a single prominent band for each target protein, except LPP3, where two additional bands were observed on higher exposure (B). LPP3: lipid phosphate phosphatase 3; SphK2: sphingosine kinase 2; SGPP1: sphingosine-1-phosphate phosphatase 1; SphK1: sphingosine kinase 1; OSCC: oral squamous cell carcinoma.

**Figure 2:**
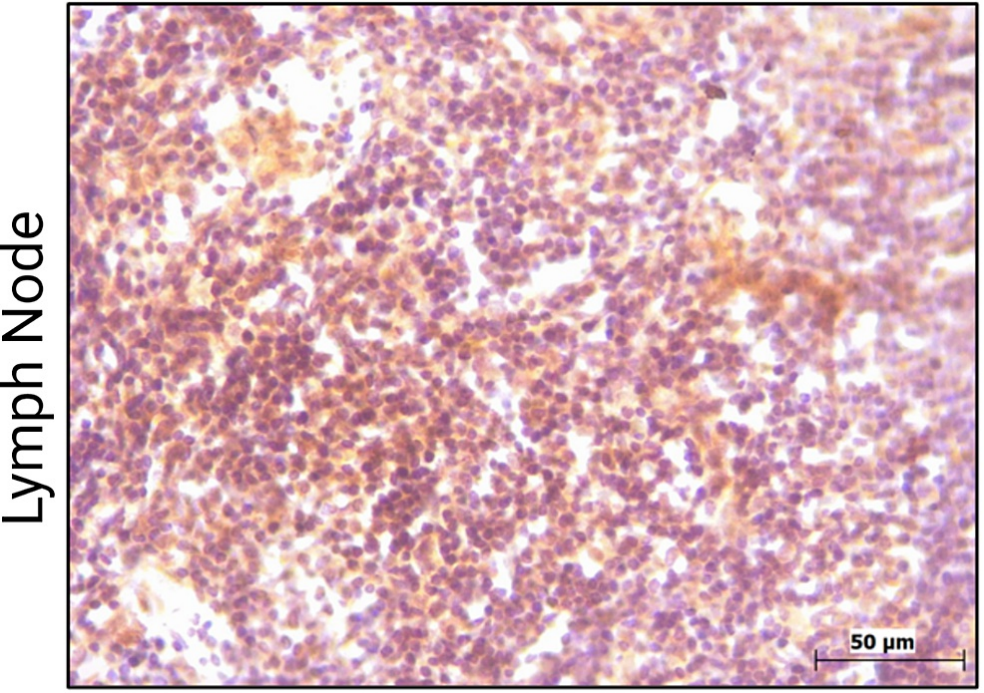
Immunohistochemistry for LPP3 in the lymph node. Diffuse cytoplasmic positivity was observed in lymphoid cells. LPP3: lipid phosphate phosphatase 3.

**Figure 3:**
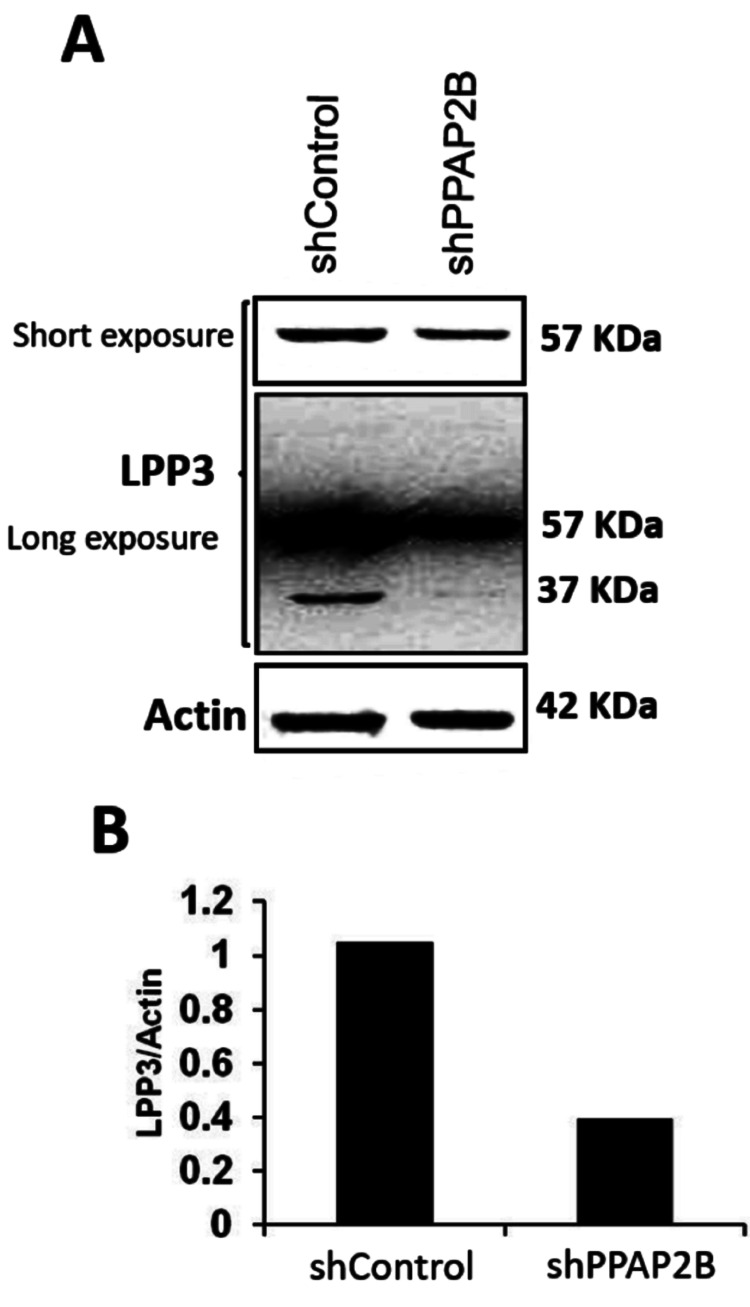
Knockdown of PPAP2B gene. (A-B) The human cervical cancer cell line, Ca Ski, was transfected with µg of shControl and pLKO.1-shPPAP2B using Fugene. Cells were harvested 60 hours post-transfection. (A) The cell lysate was subjected to western blotting; blots were incubated with LPP3 antibody or actin antibody. Signal was recorded in two exposures. Short exposure (upper panel) and long exposure (middle panel) were taken (B). Densitometric analysis of blots was performed using Image J software. Data are shown as LPP3/actin ratio. PPAP2B: phosphatidic acid phosphatase type 2B; LPP3: lipid phosphate phosphatase 3.

To validate the antibodies, immunoblotting was performed on four sets of tissue extracts (tumor and adjacent normal tissue) from OSCC patients. SphK2, LPP3, and SGPP1 detected a single prominent band in the gels (Figures 1A-1E). The molecular weight of mature LPP3 is 32 kDa, but the LPP3 antibody used in our research paper detected a prominent band at ~57 kDa (Figure 1A). On prolonged exposure of the LPP3 blot, two additional bands corresponding to ~32 kDa and ~37 kDa also appear in a few samples (Figure 1B). LPP3 is known to be heavily glycosylated, and similar bands have been detected by others [20,21]. Detection of a single prominent band by these antibodies establishes their specificity. To validate the specificity of LPP3, IHC was performed in the lymph nodes, where LPP3 is responsible for maintaining low interstitial S1P levels [22]. As shown in Figure 2, diffuse cytoplasmic positivity was observed in the lymphoid cells. Owing to the downregulation of LPP3 in tumor tissues from several cancer types [23], cancer cell lines also exhibit low expression of LPP3. We screened several cell lines to evaluate LPP3 expression. We found that Ca Ski, a cervical cancer cell line, shows an appreciable level of LPP3 (Figure 3). Thus, to validate the specificity of the LPP3 antibody, the PPAP2B gene was knocked down in Ca Ski cells using a lentiviral vector. Transient transfection with a pLKO.1-shPPAP2B decreased the expression of prominent LPP3 band to ~50% compared to control shRNA transfected cells (Figure 3A). Upon prolonged exposure, a decrease in the expression of a smaller band of ~37kDa LPP3 protein was also noted in pLKO.1-shPPAP2B cells, compared to control shRNA (Figure 3A, middle panel).

SphK1 is overexpressed in OSCC tumors

**Figure 4:**
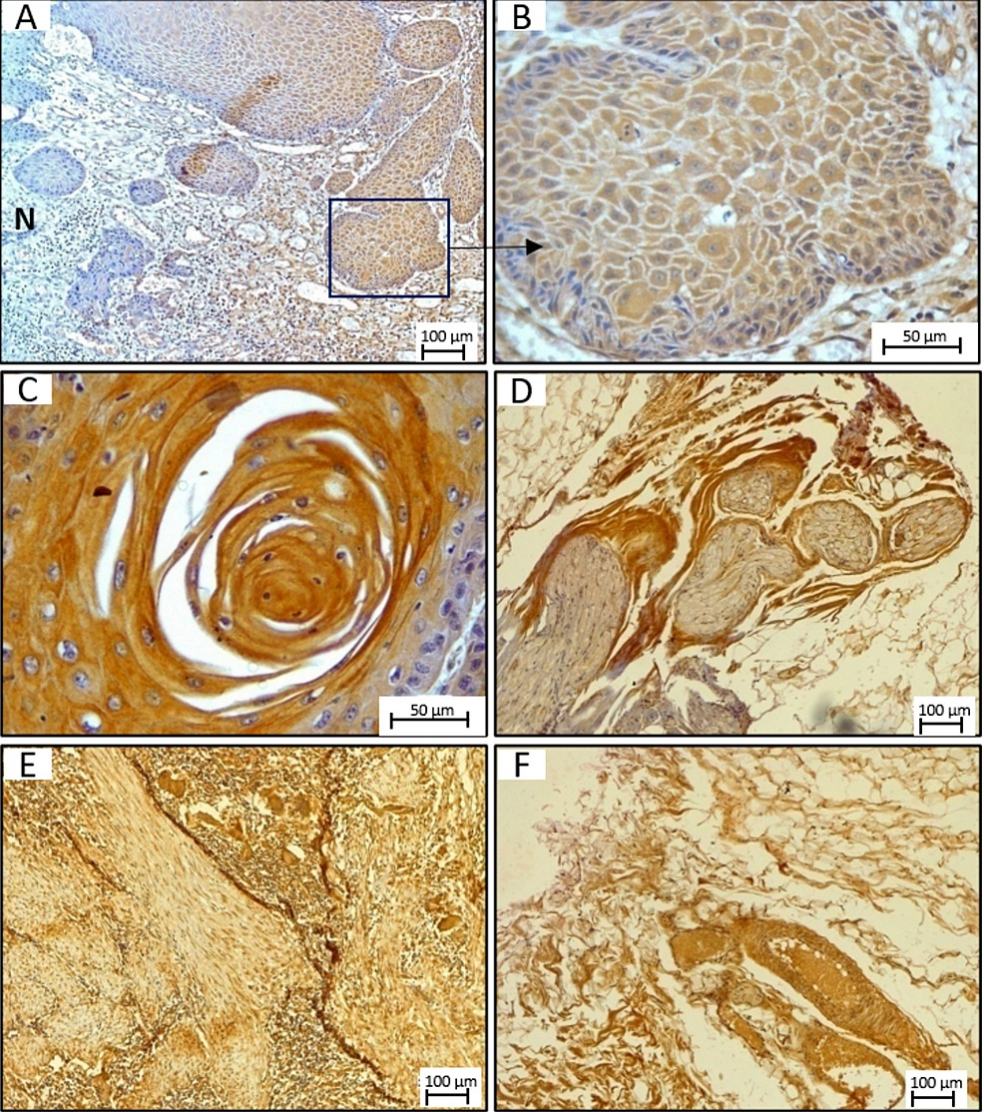
Expression of SphK1 in OSCC tumors by IHC. (A-B) Neoplastic epithelium, (C) keratin pearl, (D) neural bundle, (E) skeletal muscle, and (F) blood vessels. N is non-neoplastic mucosa. SphK1: sphingosine kinase 1; OSCC: oral squamous cell carcinoma; IHC: immunohistochemistry.

Our previous study has shown that SphK1 mRNA transcript is overexpressed in tumor tissues of OSCC patients compared to adjacent normal tissues from the same patient. To determine if it is consistent at the protein level, we performed IHC analysis on tumors from OSCC patients. Cytoplasmic positivity for SphK1 was observed in most tumor cases in malignant squamous epithelial cells (Figures 4A, 4B). IRS score ranged from 2 to 9 in the OSCC cases with positive staining. All the cases included in the study were positive for SphK1. More than half (52.2%) showed high positivity in tumor cells (Figures 4C, 4D), whereas low IRS for SphK1 was observed in 47.8% of patients. The mean IRS for SphK1 in all the cases was 4.78. Expression of SphK1 was higher in tumor cells compared to adjacent non-neoplastic cells (Figure 4A). Focal cytoplasmic positivity was also observed in the adjacent non-neoplastic epithelium in a few cases. Amongst the non-epithelial tissues, cytoplasmic positivity was also noted in the stromal cells like inflammatory cells, skeletal muscle fibers, neural bundles, and blood vessels (Figures 4D-4F).

SphK2 expression is downregulated in OSCC tumors

**Figure 5:**
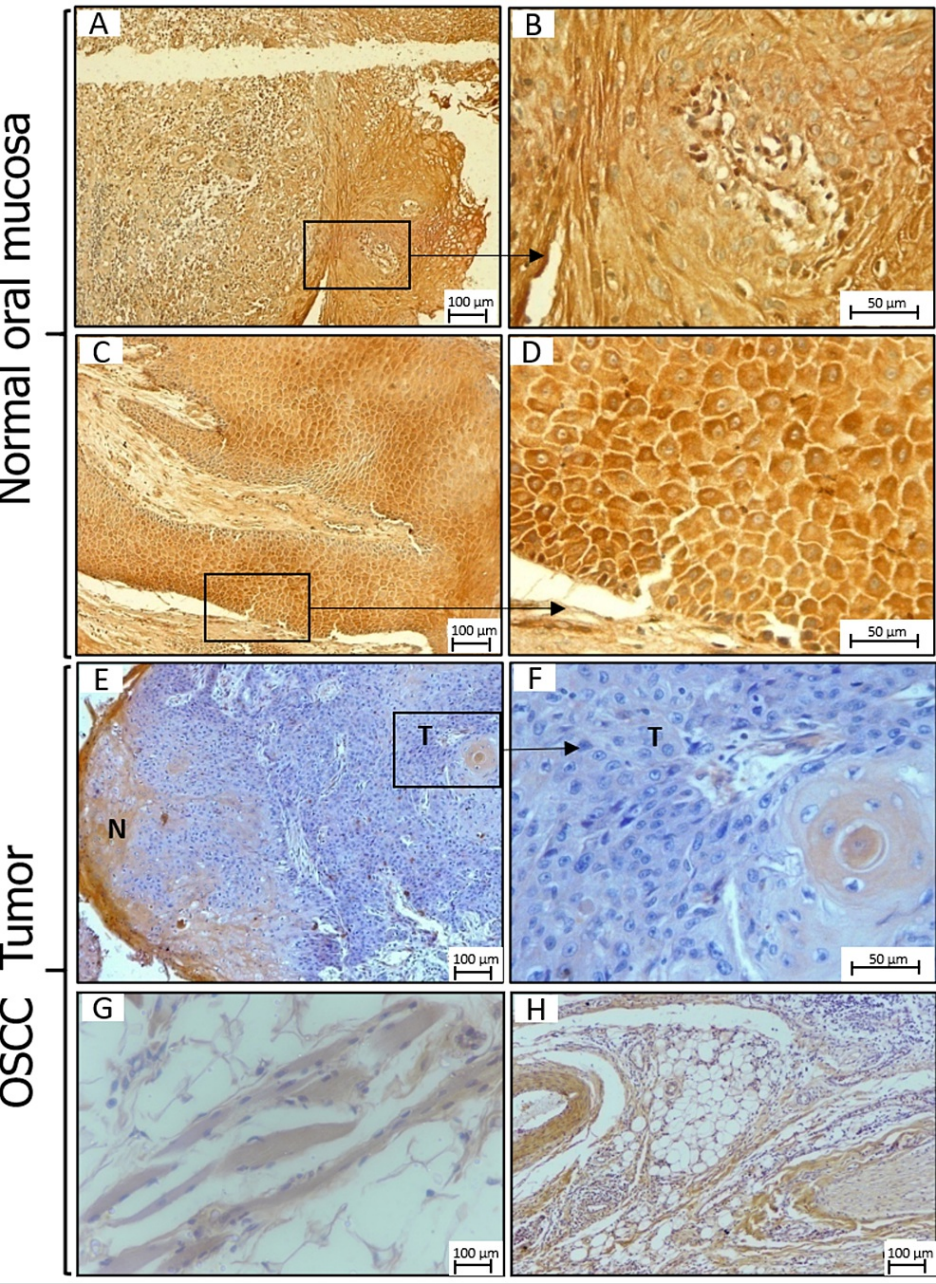
Expression of SphK2. Expression of SphK2 in the normal oral mucosa (A-D) and OSCC tumors (E-H) by IHC. (G) Skeletal muscle and (H) neurovascular bundle. N: adjacent non-neoplastic mucosa; T: neoplastic mucosa; SphK2: sphingosine kinase 2; OSCC: oral squamous cell carcinoma; IHC: immunohistochemistry.

Strong cytoplasmic positivity for SphK2 was noted in normal mucosa (Figures 5A-5D). High immunoreactivity was observed in normal mucosa. On the other hand, IRS ranged from 2 to 6 (mean IRS = 3.24) in all the OSCC cases, except one sample, where the IRS was 9.0. Although immunoreactivity was noted in almost all the cases (96%); however, more than three-fourths (80.4%) of the cases showed low expression of SphK2. High immunoreactivity was noted in 17.4% of cases, and it was mostly seen in the adjacent non-malignant cells. Expression of SphK2 in adjacent non-malignant epithelium showed moderate to strong cytoplasmic positivity, whereas low expression was noted in tumor cells (Figures 5E, 5F). The cytoplasmic activity was also recorded in skeletal muscle fibers and neurovascular bundle (Figures 5G, 5H).

SGPP1 is expressed at a low level in OSCC tumors

**Figure 6:**
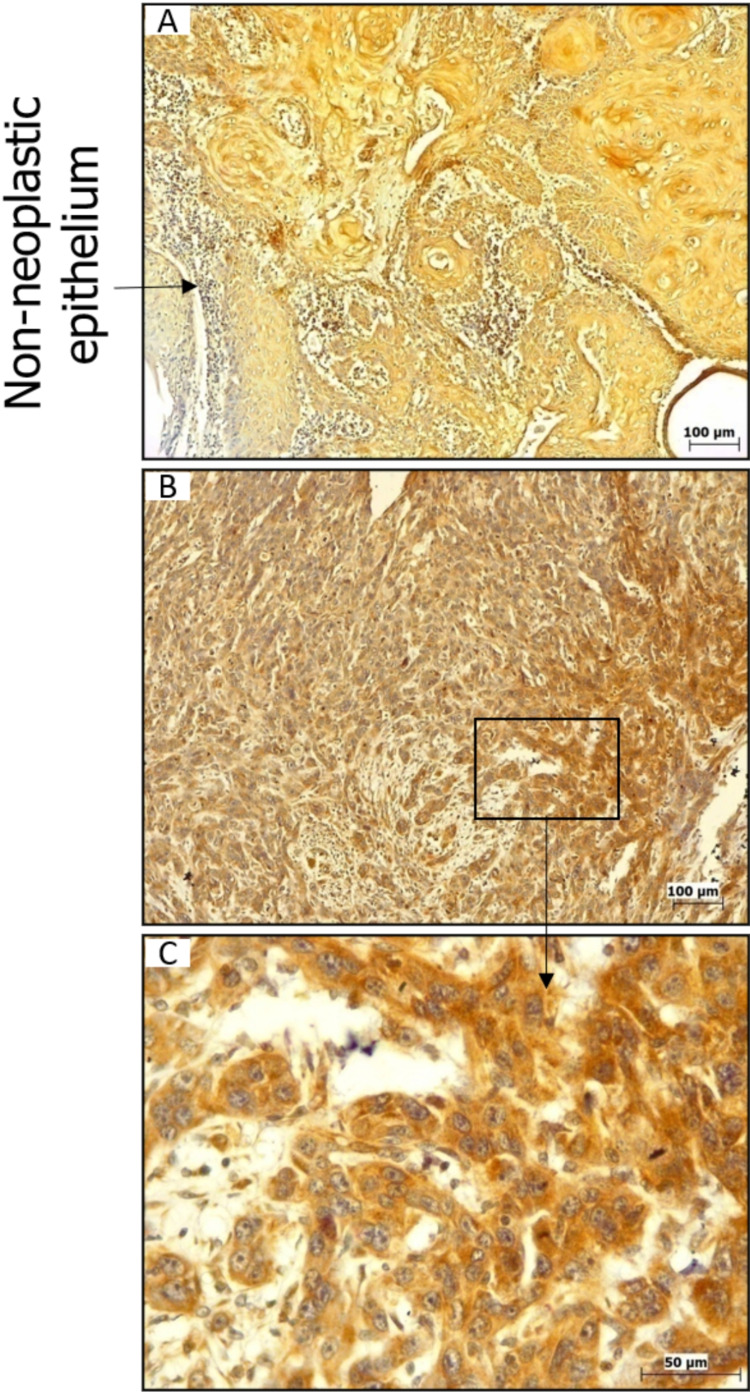
Expression of SGPP1 in OSCC tumors. Expression of SGPP1 in OSCC tumors (A-C) by IHC. SGPP1: sphingosine-1-phosphate phosphatase 1; OSCC: oral squamous cell carcinoma; IHC: immunohistochemistry.

Cytoplasmic positivity for SGPP1 was observed in the majority of the cases. IRS score ranged from 1 to 6 (mean IRS = 3.8). Although immunoreactivity was noted in almost all (89%) the cases; however, approximately two-thirds (63%) of the cases showed low expression of SGPP1, whereas 26% of cases showed high expression of SGPP1. Focal positivity was also noted in the adjacent non-neoplastic epithelium (Figure 6).

LPP3 expression is sharply downregulated in OSCC tumors

**Figure 7:**
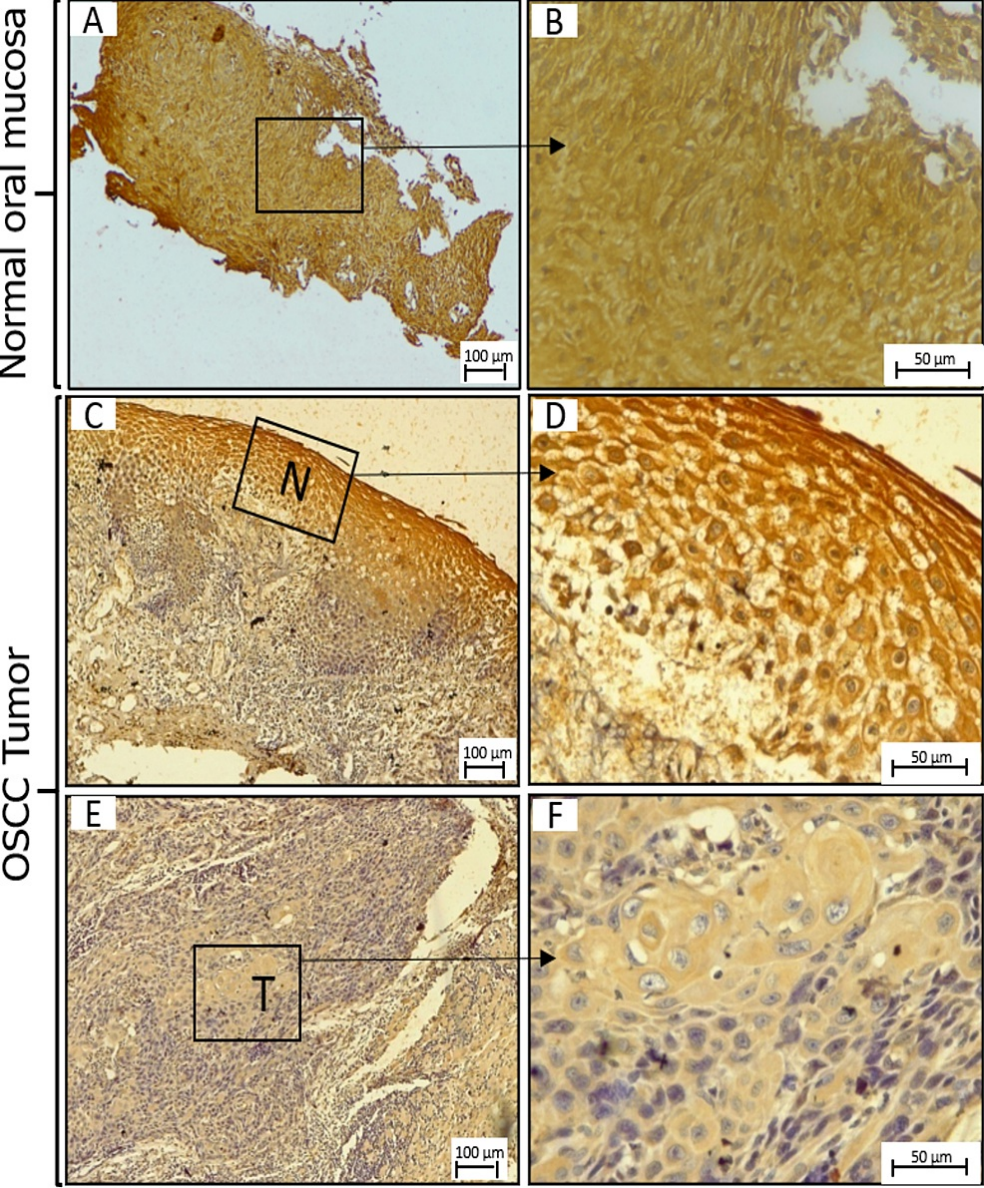
Expression of LPP3. Expression of LPP3 in the normal oral mucosa (A-B) and OSCC tumors (C-F) by IHC. N: adjacent non-neoplastic mucosa; T: neoplastic mucosa; LPP3: lipid phosphate phosphatase 3; OSCC: oral squamous cell carcinoma; IHC: immunohistochemistry.

Strong cytoplasmic and membranous immunoreactivity was noted for LPP3 in the normal oral mucosa (Figures 7A, 7B). In OSCC, 17% of cases were negative for LPP3 immunoreactivity, 58.7% of cases showed low immunoreactivity, and only 24% of cases showed high expression. The mean IRS for LPP3 in OSCC tumors was 2.89. Even in the same tissue section from OSCC patients, adjacent non-malignant cells showed high immunoreactivity compared to tumor cells (Figure 7). IRS was statistically lower in the tumor tissues compared to non-malignant tissues.

Expression of SphK2 and LPP3 is decreased in the tumor tissues than in the adjacent normal tissues

**Figure 8:**
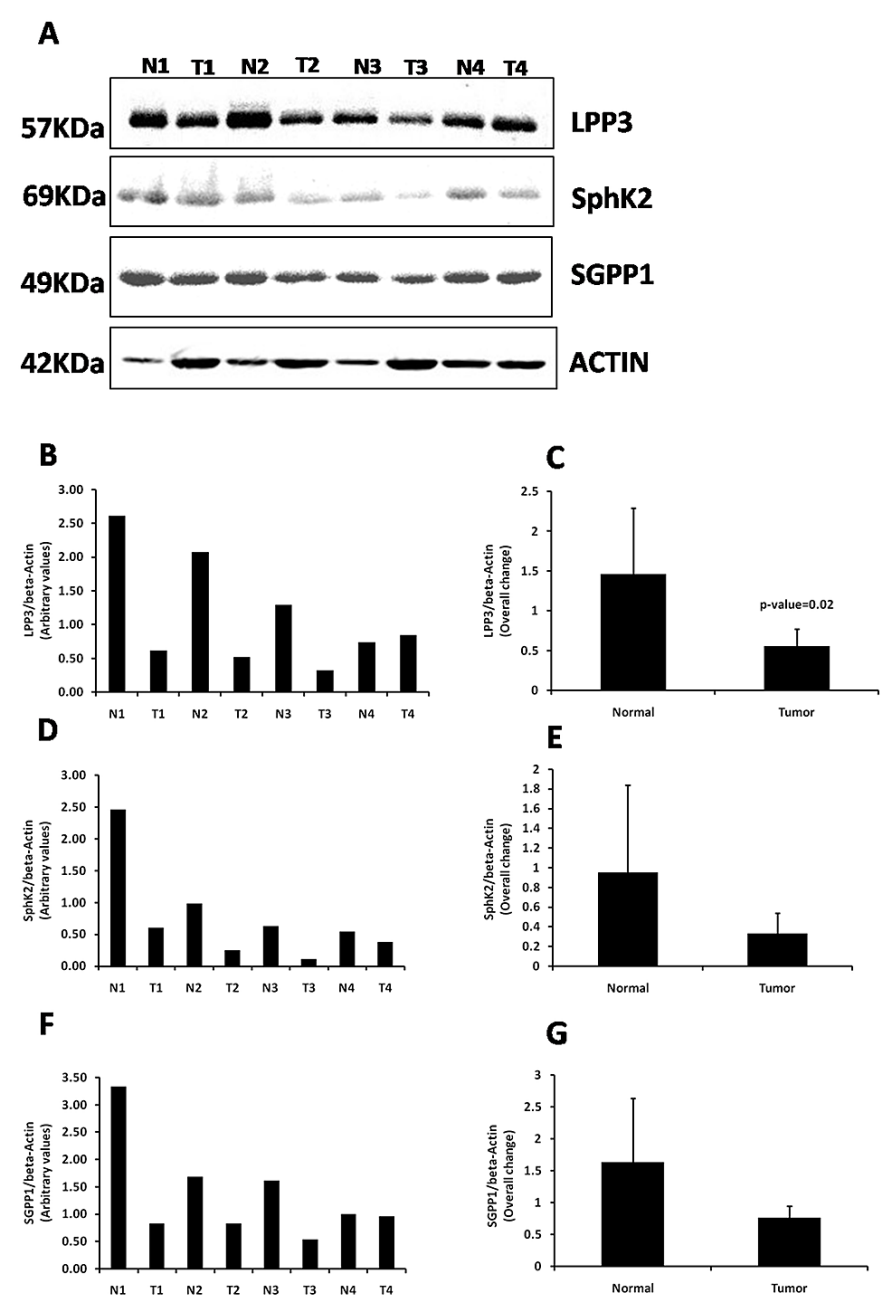
Expression of LPP3 and SphK2 decreases in the OSCC tumors. (A) Western blotting for LPP3, SphK2, SGPP1, and actin was performed in tumor tissues (T1, T2, T3, and T4) and adjacent normal tissues (N1, N2, N3, and N4) from the same patients. (B, D, and F) Densitometric analysis was performed with Image J software and the ratio of target protein/beta-actin for each sample is shown with the bar diagram. (C, E, and G) The average of target protein/beta-actin was calculated and values are shown as mean ± standard deviation. A t-test was performed to compare the difference in mean. LPP3: lipid phosphate phosphatase 3; SphK2: sphingosine kinase 2; SGPP1: sphingosine-1-phosphate phosphatase 1; OSCC: oral squamous cell carcinoma.

To validate our findings, the expression of SphK2, LPP3, and SGPP1 was analyzed by western blotting in four sets of tumor tissues and adjacent normal tissues from the same patients. As shown in Figures 5A-5G, expression of LPP3 and SphK2 was decreased in three out of four samples (Figure 8A). Quantitative analysis showed a significant decrease in LPP3/beta-actin ratio in the tumors compared to the corresponding adjacent normal tissue (Figure 8).

Association of IRS of S1P-metabolizing genes with clinicopathological features

SphK1 is overexpressed and negatively correlates with poor survival of patients [24]. However, the clinical significance of other S1P-metabolizing enzymes in OSCC is not known. Association between S1P-metabolizing enzyme expression (IRS) in tumors and clinicopathological features (age, gender, tobacco chewing, smoking status, alcohol consumption, tumor volume, tumor site, TNM staging, LNR, PNE, and LVI) was analyzed by Spearman’s rank test. In the present study, the IRS of SphK2 negatively correlated with gender (r = −0.319, p = 0.030). Female OSCC patients had lower IHC scores compared to males. IRS of SphK2 also negatively correlated with smoking status (r = −0.314, p = 0.033) (Table 1).

IRS of SGPP1 positively correlated with LNR (r = 0.313, p = 0.040). OSCC patients with low SGPP1 IRS had low LNR (<0.1). Importantly, LPP3 expression negatively correlated with TNM staging of patients (r = −0.307, p = 0.043) and LVI (r = −0.530, p < 0.001). OSCC patients presented in the advanced stages (III + IV) of the disease had low IRS for LPP3 (Table 2).

**Table 2:** Frequency of SGPP1 and LPP3 IRS score. The frequency of SGPP1 and LPP3 has been calculated with different clinicopathological attributes of OSCC patients. Tumor-node-metastasis (TNM) staging has been grouped into two (Groups I and II), whereas histology has been divided into well-differentiated squamous cell carcinoma (WDSCC), moderately differentiated squamous cell carcinoma (MDSCC), and poorly differentiated squamous cell carcinoma (PDSCC). SGPP1: sphingosine-1-phosphate phosphatase 1; LPP3: lipid phosphate phosphatase 3; IRS: immunoreactivity score; OSCC: oral squamous cell carcinoma

S. No.	Groups	Total (n = 46)	SGPP1 expression		LPP3 expression	
			Low	High	Low	High
1	Age					
	<50 years	27	20 (74.1 %)	07 (25.9%)	22 (81.5%)	05 (18.5%)
	>50 years	19	14 (73.7%)	05 (26.3%)	13 (68.4%)	06 (13.6%)
	P-value		NS			
2	Sex					
	Male	31	23 (74.2%)	08 (25.8%)	24 (77.4%)	07 (22.6%)
	Female	15	11 (73.3%)	04 (26.7%)	11 (73.3%)	04 (26.7%)
	P-value		NS			
3	Tumor size					
	T1-T2	13	09 (69.2%)	04 (30.8%)	08 (61.5%)	05 (38.5%)
	T3-T4	31	23 (74.2%)	08 (25.8%)	26 (83.9%)	05 (16.1%)
	P-value		NS			
4	Lymph nodes					
	N0	19	15 (78.9%)	04 (21.1%)	13 (68.4%)	06 (31.6%)
	N1-N3	25	17 (68.0%)	08 (32.0%)	21 (84.0%)	04 (16.0%)
	P-value		NS			
5	Distant metastasis					
	M0	44	32 (72.7%)	12 (27.3%)	34 (77.3%)	10 (22.7%)
	M1	00	00	00	00	00
	P-value		NS			
6	Smoking status					
	Yes	10	06 (60.0%)	04 (40.0%)	06 (60.0%)	04 (40.0%)
	No	36	28 (77.8%)	08 (22.2%)	29 (80.5%)	07 (19.5%)
	P-value		NS			
7	Alcohol status					
	Yes	10	05 (50.0%)	05 (50.0%)	08 (80.0%)	02 (20.0%)
	No	32	25 (78.1%)	07 (21.9%)	24 (75.0%)	08 (25.0%)
	P-value		NS			
8	Tumor volume					
	Less than 4 cm3	20	16 (80.0%)	04 (20.0%)	14 (70.0%)	06 (30.0%)
	More than 4 cm3	24	16 (66.7%)	08 (33.3%)	20 (83.3%)	04 (16.7%)
	P-value		NS			
9	Peri-nodal extension (PNE) involvement					
	Yes	11	09 (81.8%)	02 (18.2%)	06 (54.5%)	05 (45.5%)
	No	33	23 (69.7%)	10 (30.3%)	28 (84.8%)	05 (15.2%)
	P-value		NS			
10	Lymphovascular invasion (LVI)					
	Yes	03	02 (66.7%)	01 (33.3%)	00	03 (100.0%)
	No	38	27 (71.1%)	11 (28.9%)	32 (84.2%)	06 (15.8%)
	P-value		NS			
11	Lymph-node ratio (LNR)					
	Less than <0.1	29	24 (82.8%)	05 (17.2%)	25 (86.2%)	04 (13.8%)
	More than >0.1	15	08 (53.3%)	07 (46.7%)	09 (60.0%)	06 (40.0%)
	P-value		0.038		0.049	
12	TNM staging					
	Group 1 (Stage I + II)	08	06 (75.0%)	02 (25.0%)	04 (50.0%)	04 (50.0%)
	Group 2 (Stage III + IV)	36	26 (72.2%)	10 (27.8%)	30 (83.3%)	06 (16.7%)
	P-value		NS		0.042	
13	Tumor site					
	Buccal mucosa	23	19 (82.6%)	04 (17.4%)	17 (73.9%)	06 (26.1%)
	Tongue	12	08 (66.7%)	04 (33.3%)	08 (66.7%)	04 (33.3%)
	Alveolus	7	04 (57.1%)	03 (42.9%)	07 (100.0%)	00
	Lip	4	03 (75.0%)	01 (25.0%)	03 (75.0%)	01 (25.0%)
	P-value		NS			
14	Histology					
	WDSCC	38	28 (73.7%)	10 (26.3%)	30 (78.9%)	08 (21.1%)
	MDSCC	08	06 (75.0%)	02 (25.0%)	05 (62.5%)	03 (37.5%)
	PDSCC	00	00			
	P-value		NS			

Clinicopathological features as an independent predictor of expression of S1P-metabolizing enzymes

To find out whether clinicopathological attributes (age, gender, tobacco chewing, smoking, alcohol consumption, tumor volume, TNM staging, PNE, LVI, and LNR) of OSCC patients could serve as an independent predictor of IRS in protein expression of S1P-metabolizing genes, multivariate linear regression analysis was performed. Tumor size (standardized coefficient β = −0.562, p = 0.035), gender (standardized coefficient β = −0.444, p = 0.040), and smoking status (standardized coefficient β = −2.240, p < 0.001) were found to be an independent negative predictor of SphK2 expression. LVI was found to be a strong negative predictor of LPP3 expression (standardized coefficient β = −0.653, p =0.002) (Table 2).

S1P-metabolizing gene expression as a predictor for clinicopathological features

To determine whether protein expression of S1P-metabolizing enzymes in tumors could determine clinicopathological features (tumor volume, TNM staging, PNE, LNR, and LVI) of OSCC patients, multivariate linear regression analysis was done. The IRS of LPP3 in tumors was found to be an independent predictor of TNM staging (standardized coefficient β = −0.364, p = 0.030), PNE (standardized coefficient β = −0.440, p = 0.009), LVI (standardized coefficient β = −0.614, p < 0.001), and LNR (standardized coefficient β = 0.336, p = 0.039) (Table 2).

LPP3 expression correlates with infiltration of immune cells in HNSCC tumors

The role of the S1P-metabolizing enzyme and S1P receptors in the regulation of infiltration of immune cells to the tumor stroma in HNSCC is not known. Correlation between TIICs and the IRS of S1P-metabolizing enzymes was determined by Spearman’s rank test. A trend of positive correlation (r = 0.22) was obtained between the number of TIICs in the OSCC tumors and IRS of LPP3, which did not reach statistical significance due to the low sample size.

**Figure 9:**
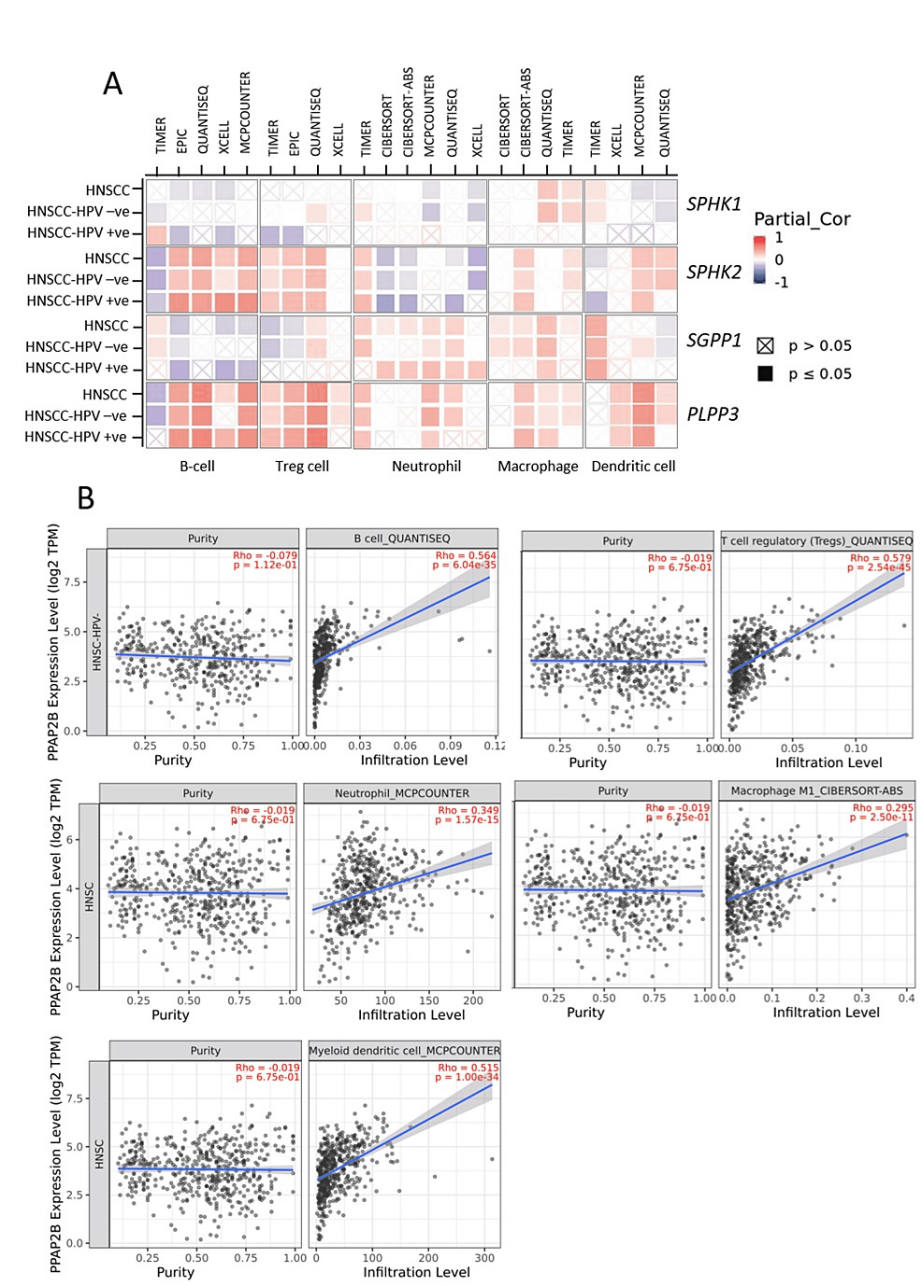
Correlation of mRNA expression of S1P-metabolizing enzymes with immune infiltration level. Correlation of mRNA expression of S1P-metabolizing enzymes with immune infiltration level in HNSCC (N = 522), HPV-negative (N = 422), and HPV-positive (N = 98) patients was determined using Tumor Immune Estimation Resource 2.0 (TIMER 2.0). Association between mRNA expression of S1P-metabolizing enzymes and tumor-infiltrating immune cells was analyzed by the "Immune" module of TIMER 2.0 (A). A heat map was generated for showing the positive, negative, or no correlation between gene expression and infiltration level of immune cells (B). Representative dot plots were drafted between PPAP2B expression Log2 TPM (transcript per million) and infiltration level. S1P: sphingosine-1-phosphate; SphK1: sphingosine kinase 1; SphK2: sphingosine kinase 2; SGPP1: sphingosine-1-phosphate phosphatase 1; PLPP3: phospholipid phosphatase 3; HNSCC: head and neck squamous cell carcinoma; HPV: human papillomavirus; PPAP2B: phosphatidic acid phosphatase type 2B.

To further analyze the association between the expression of S1P-metabolizing genes and TIICs, TCGA data were analyzed by TIMER 2.0, a web-based server. In TIMER 2.0, correlation is determined by Spearman’s rank test, and a heat map is generated based on positive, negative, or no significant correlation obtained from different algorithms [18]. As shown in Figure 9A, LPP3 showed moderate to a high positive correlation with the infiltration level of B cells, Tregs, neutrophils, M1 macrophages, and DC in HNSCC tumors. The correlation was consistent in both the subtypes HPV negative and HPV positive HNSCC tumors with different algorithms (Figure 9A), and representative plots are shown in Figure 9B. LPP3 did not show a consistent correlation with infiltration levels of CD4+, CD8+ T cells, and NK cells. SphK2 expression showed a consistent positive correlation with the infiltration level of B cells and Tregs, whereas SGPP1 showed a consistent correlation with the infiltration level of neutrophils and macrophages in HNSCC tumors (Figure 9A). However, SphK1 expression showed a negative correlation with the infiltration level of B cells in the HNSCC tumors, and no consistent correlation was observed with the other cell types (Figure 9A).

**Figure 10:**
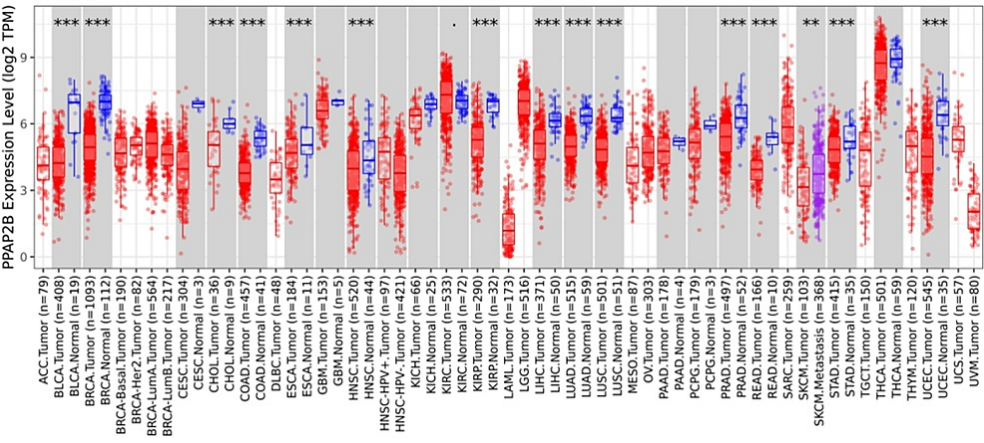
Distributions of PPAP2B gene expression levels. Distributions of PPAP2B gene expression levels are displayed using box plots. The statistical significance computed by differential analysis (edgeR) on RNA-Seq raw counts is annotated by the number of stars (*: p-value < 0.05; **: p-value < 0.01; ***: p-value < 0.001). PPAP2B: phosphatidic acid phosphatase type 2B; TPM: transcript per million.

We also analyzed the differential expression of LPP3 (PPAP2B) across all TCGA tumors using the Gene_DE module. As shown in Figure 10, besides HNSCC, the expression of PPAP2B is decreased significantly in several cancer types, including the cancer of the urinary bladder, colon, esophagus, liver, pancreas, cholangiocarcinoma, lung adenocarcinoma, and lung squamous cell carcinoma.

## Discussion

OSCC is the most common cancer in the Indian subcontinent, and it is one of the leading causes of cancer-related deaths among Indian men [2]. S1P signaling has been shown to play a crucial role in several processes integral to carcinogenesis, including angiogenesis, invasion, metastasis, and development of resistance to chemo-radiation [7]. SphK1, a key enzyme involved in S1P synthesis, promotes tumor progression, invasion, metastasis, and chemoresistance in HNSCC, and its expression level correlates with patient survival [24-26]. Previously, we had reported that mRNA expression of SphK1 is overexpressed in the tumors of 70% of OSCC patients. There was an overall 73% increase in the SphK1 expression in tumors than adjacent normal tissues [14]. Consistent with our quantitative reverse transcription-polymerase chain reaction (qRT-PCR) data, we found the high expression of SphK1 by IHC in malignant epithelial cells, keratin pearl, and stromal cells of OSCC tumors. These findings were in accordance with a previously published study [24]. SphK1 expression has been shown to associate with the invasiveness of OSCC tumors and the poor prognosis of patients [24].

Here, we have reported, for the first time, the expression of SphK2 by IHC in OSCC tumors. Low immunoreactivity seen in OSCC tumors is consistent with our qRT-PCR data, where a decrease in SphK2 mRNA expression was noted in 71% of OSCC patients [14]. In multivariate linear regression analysis, tumor size was an independent negative predictor of SphK2 expression. In our study, we have found nuclear as well cytoplasmic immunoreactivity for SphK2, which is mainly a nuclear protein. However, a previous study has shown that phosphorylation of Sphk2 by protein kinase D leads to its nuclear export [27]. This might explain nuclear as well as cytoplasmic immunoreactivity for SphK2. In corroboration of our study, SphK2-deficient mice produced high-grade adenomas and large tumors in a mouse model of colitis-associated cancer [28]. On the contrary, high expression of SphK2 has been reported in other cancer types, including breast [29], lung [30], and colon cancers [31]. Though both the isoforms of SphKs catalyze the reaction and form S1P; however, their roles differ in cellular functions [32]. Initial studies conducted to characterize SphK2 functions have revealed that it can promote cell cycle arrest and apoptosis [33].

It has been suggested that depending on the intracellular localization, SphK2 can play pro-apoptotic or anti-apoptotic functions [32]. Several mechanisms have been proposed to explain the pro-apoptotic functions of SphK2. Spiegel and colleagues suggested that SphK2 possesses a functional BH3 domain and may contribute to apoptosis by sequestering pro-survival B-cell lymphoma 2 (Bcl-2) family proteins [33]. SphK2 has also been shown to localize in mitochondria, where it directly binds with pro-apoptotic Bcl-2 family member BAK and induces the latter’s activation [34]. Thus, low expression of SphK2 in oral squamous cells may suppress apoptosis, thereby contributing to neoplasia.

Our study found SGPP1 immunoreactivity in 89% of cases of OSCC, albeit it was low in two-thirds of cases. Decreased expression of SGPP1 enhances cell migration and resistance to DNA damaging drugs [35]. Further, downregulation in SGPP1 causes endoplasmic reticulum (ER) stress-induced autophagy [36]. Thus, increased levels of SGPP1 in OSCC tumors may protect the squamous cells against ER stress and autophagy, thereby promoting oral carcinogenesis. Moreover, loss of SGPP1 in mice has been shown to induce keratinocyte differentiation [37]. Thus, increased SGPP1 expression may cause dedifferentiation of oral epithelium and may contribute to oral carcinogenesis.

This is the first report showing the expression of LPP3 by IHC in OSCC tumor tissues. In the present study, we have demonstrated that LPP3 is strongly expressed in normal oral mucosa, whereas OSCC tumors show low (~60% cases) or loss (17% cases) of LPP3 expression. Together, we can conclude that protein expression of LPP3 is suppressed in OSCC tumors, which is in corroboration with our mRNA expression data and Oncomine data analysis published recently [14,23]. Thus, the expression of LPP3 in OSCC is dysregulated at the transcriptional level; however, the mechanism of dysregulated expression of LPP3 in OSCC is not known. In addition to HNSCC, downregulation of LPP3 in the tumors has been shown in several cancer types including, colon, breast, and lung cancers [38,39]. LPP3 has been shown to regulate angiogenesis and embryonic development [40]. In bivariate analysis, LPP3 negatively correlates with TNM staging and LVI of patients.

Further, in multivariate linear regression analysis, LPP3 expression was an independent negative predictor of TNM staging, LVI, PNE, and a positive predictor of LNR. Previously, we have also reported a negative correlation between tumor volume and expression of LPP3 [14], thus suggesting LPP3 might play an important role in tumorigenesis. Hypoxia is the usual characteristic of advanced-stage solid tumors. Indeed, hypoxia enables various events in the TME that results in the expansion of aggressive clones from heterogeneous tumor cells. In our study, LPP3 expression was negatively correlated with advanced stages of cancer. Towards this, recent work by Harper et al. suggested that LPP3 downregulation is mediated by hypoxia [41]. Therefore, hypoxia can drive tumor invasion and metastasis by modulating the expression LPP3. Importantly, LPP3 has been shown to decrease the growth, survival, and tumorigenesis of ovarian cancer cells [42]. LPP3 is a broad specificity plasma membrane-bound lipid phosphate phosphatase that can dephosphorylate various lipid phosphates, including phosphatidate, lysophosphatidic acid (LPA), S1P, ceramide-1-phosphate, and diacylglycerol pyrophosphate present in the extracellular milieu [40]. Downregulation of LPP3 in OSCC may lead to accumulation of S1P in squamous epithelial cells, which may promote carcinogenesis by various mechanisms such as regulating cell proliferation, angiogenesis, and metastasis. Thus, LPPs may negatively regulate oral carcinogenesis. Whether downregulation of LPP3 in OSCC results in the accumulation of other lipid phosphates is not known. Notably, LPA present in saliva has also been related to OSCC cell migration and invasiveness [43,44]. On the contrary, higher expression of LPP3 has been reported in the primary gliomas and glioblastoma cell lines [45]. In that study, the authors showed that LPP3 promotes tumor growth through the beta-catenin pathway [46]. The reason for the differential effect of LPP3 in the different cancer models is not known.

Tumor-associated macrophages are broadly categorized into two types: M1-like macrophages and M2-like macrophages. M1-like macrophages are pro-inflammatory, exert antitumor effects, and are associated with a better prognosis of the patients [47]. Consistently, in our study, we found a positive correlation between LPP3 expression and the presence of B cells, Tregs, neutrophils, M1 type of macrophages, and DCs in the TME of HNSCC tumors. We found a similar correlation between LPP3 expression and TIICs in lung adenocarcinoma [47]. Furthermore, significantly higher expression of B cell-related genes and higher densities of CD20+ B cells in HPV+ OSCC samples have been reported, associated with a better prognosis of the patients [17]. S1P gradient and S1P receptor axis represent an obligatory signal for the trafficking of immune cells [7]. Thus, downregulation of LPP3 in the tumors may destroy the S1P gradient in TME and affect the recruitment of TIICs in HNSCC tumors. LPPs also dephosphorylate LPA; it has also been shown that LPA and autotaxin, an LPA synthesizing enzyme, play a role in steady-state lymphocyte homing, but their impact on the regulation of adaptive immunity needs further understanding [23,48]. This is an area of future investigation in the context of the expanding role of immunotherapy in HNC. Further, the mechanism behind the dysregulation of LPP3 has not been determined yet, and whether the downregulation of LPP3 results in the elevation of S1P or LPA is also not known. Moreover, whether the downregulation of LPP3 directly controls tumorigenesis needs to be explored.

## Conclusions

This is the first report showing the expression of LPP3 by IHC and western blotting in OSCC tumor tissues. Here, we demonstrate that OSCC tumors have low expression of SphK2 and LPP3, compared to the normal oral epithelium. However, the mechanism of dysregulated expression of LPP3 and SphK2 in OSCC is not known. Furthermore, LPP3 negatively correlates with the TNM staging of patients. Downregulation of LPP3 may result in increased S1P levels, resulting in tumor progression. However, the causal link has not been determined. We also concluded that downregulation of LPP3 in the tumors may destroy the S1P gradient in TME and affect the recruitment of TIICs in HNSCC tumors.

## References

[1] Sung H, Ferlay J, Siegel RL, Laversanne M, Soerjomataram I, Jemal A, Bray F (2021). Global Cancer Statistics 2020: GLOBOCAN estimates of incidence and mortality worldwide for 36 cancers in 185 countries. CA Cancer J Clin.

[2] Mallath MK, Taylor DG, Badwe RA (2014). The growing burden of cancer in India: epidemiology and social context. Lancet Oncol.

[3] Jain D, Dravid C, Singla A, Kumari S, Grover RK (2019). Comparison of the seventh and eighth editions of the American Joint Committee on Cancer pT and pN classifications as predictors of survival in patients with oral squamous cell carcinoma. Am J Clin Pathol.

[4] Shaib W, Kono S, Saba N (2012). Antiepidermal growth factor receptor therapy in squamous cell carcinoma of the head and neck. J Oncol.

[5] Kumar A, Saba JD (2018). Sphingosine-1-phosphate. Encyclopedia of Signaling Molecules.

[6] Nagahashi M, Abe M, Sakimura K, Takabe K, Wakai T (2018). The role of sphingosine-1-phosphate in inflammation and cancer progression. Cancer Sci.

[7] Ogretmen B (2018). Sphingolipid metabolism in cancer signalling and therapy. Nat Rev Cancer.

[8] Hatoum D, Haddadi N, Lin Y, Nassif NT, McGowan EM (2017). Mammalian sphingosine kinase (SphK) isoenzymes and isoform expression: challenges for SphK as an oncotarget. Oncotarget.

[9] Le Stunff H, Galve-Roperh I, Peterson C, Milstien S, Spiegel S (2002). Sphingosine-1-phosphate phosphohydrolase in regulation of sphingolipid metabolism and apoptosis. J Cell Biol.

[10] Ogawa C, Kihara A, Gokoh M, Igarashi Y (2003). Identification and characterization of a novel human sphingosine-1-phosphate phosphohydrolase, hSPP2. J Biol Chem.

[11] Brindley DN, Pilquil C (2009). Lipid phosphate phosphatases and signaling. J Lipid Res.

[12] Benesch MG, Tang X, Venkatraman G, Bekele RT, Brindley DN (2016). Recent advances in targeting the autotaxin-lysophosphatidate-lipid phosphate phosphatase axis in vivo. J Biomed Res.

[13] Neubauer HA, Pham DH, Zebol JR (2016). An oncogenic role for sphingosine kinase 2. Oncotarget.

[14] Vishwakarma S, Agarwal R, Goel SK (2017). Altered expression of sphingosine-1-phosphate metabolizing enzymes in oral cancer correlate with clinicopathological attributes. Cancer Invest.

[15] Chen SM, Krinsky AL, Woolaver RA, Wang X, Chen Z, Wang JH (2020). Tumor immune microenvironment in head and neck cancers. Mol Carcinog.

[16] Lei Y, Xie Y, Tan YS, Prince ME, Moyer JS, Nör J, Wolf GT (2016). Telltale tumor infiltrating lymphocytes (TIL) in oral, head & neck cancer. Oral Oncol.

[17] Hladíková K, Koucký V, Bouček J (2019). Tumor-infiltrating B cells affect the progression of oropharyngeal squamous cell carcinoma via cell-to-cell interactions with CD8+ T cells. J Immunother Cancer.

[18] Li T, Fan J, Wang B (2017). TIMER: a web server for comprehensive analysis of tumor-infiltrating immune cells. Cancer Res.

[19] Li T, Fu J, Zeng Z (2020). TIMER2.0 for analysis of tumor-infiltrating immune cells. Nucleic Acids Res.

[20] Waggoner DW, Martin A, Dewald J, Gómez-Muñoz A, Brindley DN (1995). Purification and characterization of a novel plasma membrane phosphatidate phosphohydrolase from rat liver. J Biol Chem.

[21] Wary KK, Humtsoe JO (2005). Anti-lipid phosphate phosphohydrolase-3 (LPP3) antibody inhibits bFGF- and VEGF-induced capillary morphogenesis of endothelial cells. Cell Commun Signal.

[22] Simmons S, Sasaki N, Umemoto E (2019). High-endothelial cell-derived S1P regulates dendritic cell localization and vascular integrity in the lymph node. Elife.

[23] Tang X, Brindley DN (2020). Lipid phosphate phosphatases and cancer. Biomolecules.

[24] Kato K, Shimasaki M, Kato T, Segami N, Ueda Y (2018). Expression of sphingosine kinase-1 is associated with invasiveness and poor prognosis of oral squamous cell carcinoma. Anticancer Res.

[25] Tamashiro PM, Furuya H, Shimizu Y, Kawamori T (2014). Sphingosine kinase 1 mediates head & neck squamous cell carcinoma invasion through sphingosine 1-phosphate receptor 1. Cancer Cell Int.

[26] Tamashiro PM, Furuya H, Shimizu Y, Iino K, Kawamori T (2013). The impact of sphingosine kinase-1 in head and neck cancer. Biomolecules.

[27] Ding G, Sonoda H, Yu H (2007). Protein kinase D-mediated phosphorylation and nuclear export of sphingosine kinase 2. J Biol Chem.

[28] Liang J, Nagahashi M, Kim EY (2013). Sphingosine-1-phosphate links persistent STAT3 activation, chronic intestinal inflammation, and development of colitis-associated cancer. Cancer Cell.

[29] Antoon JW, White MD, Slaughter EM (2011). Targeting NFĸB mediated breast cancer chemoresistance through selective inhibition of sphingosine kinase-2. Cancer Biol Ther.

[30] Liu W, Ning J, Li C, Hu J, Meng Q, Lu H, Cai L (2016). Overexpression of Sphk2 is associated with gefitinib resistance in non-small cell lung cancer. Tumour Biol.

[31] Shi WN, Cui SX, Song ZY (2017). Overexpression of SphK2 contributes to ATRA resistance in colon cancer through rapid degradation of cytoplasmic RXRα by K48/K63-linked polyubiquitination. Oncotarget.

[32] Neubauer H, Pitson S (2018). Sphingosine kinase 2 (SPHK2). Encyclopedia of Signaling Molecules.

[33] Liu H, Toman RE, Goparaju SK (2003). Sphingosine kinase type 2 is a putative BH3-only protein that induces apoptosis. J Biol Chem.

[34] Chipuk JE, McStay GP, Bharti A (2012). Sphingolipid metabolism cooperates with BAK and BAX to promote the mitochondrial pathway of apoptosis. Cell.

[35] Le Stunff H, Mikami A, Giussani P, Hobson JP, Jolly PS, Milstien S, Spiegel S (2004). Role of sphingosine-1-phosphate phosphatase 1 in epidermal growth factor-induced chemotaxis. J Biol Chem.

[36] Lépine S, Allegood JC, Park M, Dent P, Milstien S, Spiegel S (2011). Sphingosine-1-phosphate phosphohydrolase-1 regulates ER stress-induced autophagy. Cell Death Differ.

[37] Allende ML, Sipe LM, Tuymetova G, Wilson-Henjum KL, Chen W, Proia RL (2013). Sphingosine-1-phosphate phosphatase 1 regulates keratinocyte differentiation and epidermal homeostasis. J Biol Chem.

[38] Tang X, McMullen TP, Brindley DN (2019). Increasing the low lipid phosphate phosphatase 1 activity in breast cancer cells decreases transcription by AP-1 and expressions of matrix metalloproteinases and cyclin D1/D3. Theranostics.

[39] Leung DW, Tompkins CK, White T (1998). Molecular cloning of two alternatively spliced forms of human phosphatidic acid phosphatase cDNAs that are differentially expressed in normal and tumor cells. DNA Cell Biol.

[40] Tang X, Benesch MG, Brindley DN (2015). Lipid phosphate phosphatases and their roles in mammalian physiology and pathology. J Lipid Res.

[41] Harper K, Brochu-Gaudreau K, Saucier C, Dubois CM (2019). Hypoxia downregulates LPP3 and promotes the spatial segregation of ATX and LPP1 during cancer cell invasion. Cancers (Basel).

[42] Tanyi JL, Morris AJ, Wolf JK (2003). The human lipid phosphate phosphatase-3 decreases the growth, survival, and tumorigenesis of ovarian cancer cells: validation of the lysophosphatidic acid signaling cascade as a target for therapy in ovarian cancer. Cancer Res.

[43] Xu M, Yin H, Cai Y (2019). Lysophosphatidic acid induces integrin β6 expression in human oral squamous cell carcinomas cells via LPAR1 coupling to Gαi and downstream SMAD3 and ETS-1 activation. Cell Signal.

[44] Brusevold IJ, Tveteraas IH, Aasrum M, Ødegård J, Sandnes DL, Christoffersen T (2014). Role of LPAR3, PKC and EGFR in LPA-induced cell migration in oral squamous carcinoma cells. BMC Cancer.

[45] Chatterjee I, Humtsoe JO, Kohler EE, Sorio C, Wary KK (2011). Lipid phosphate phosphatase-3 regulates tumor growth via β-catenin and CYCLIN-D1 signaling. Mol Cancer.

[46] Kumar AT, Knops A, Swendseid B (2019). Prognostic significance of tumor-associated macrophage content in head and neck squamous cell carcinoma: a meta-analysis. Front Oncol.

[47] Nema R, Shrivastava A, Kumar A (2021). Prognostic role of lipid phosphate phosphatases in non-smoker, lung adenocarcinoma patients. Comput Biol Med.

[48] Lee SC, Dacheux MA, Norman DD, Balázs L, Torres RM, Augelli-Szafran CE, Tigyi GJ (2020). Regulation of tumor immunity by lysophosphatidic acid. Cancers.

